# Wide-gamut lasing from a single organic chromophore

**DOI:** 10.1038/s41377-018-0102-1

**Published:** 2018-12-05

**Authors:** S. Lane, S. Vagin, H. Wang, W. R. Heinz, W. Morrish, Y. Zhao, B. Rieger, A. Meldrum

**Affiliations:** 1 Department of Physics, University of Alberta, Edmonton, AB T6G2E1 Canada; 2 Department of Chemistry, Technical University of Munich, Lichtenbergstraße 4, 85747 Garching bei München, Germany

## Abstract

The development of wideband lasing media has deep implications for imaging, sensing, and display technologies. We show that a single chromophore can be engineered to feature wide-gamut fluorescence and lasing throughout the entire visible spectrum and beyond. This exceptional color tuning demonstrates a chemically controlled paradigm for light emission applications with precise color management. Achieving such extensive color control requires a molecular blueprint that yields a high quantum efficiency and a high solubility in a wide variety of liquids and solids while featuring a heterocyclic structure with good steric access to the lone pair electrons. With these requirements in mind, we designed a lasing chromophore that encloses a lasing color space twice as large as the sRGB benchmark. This record degree of color tuning can in principle be adapted to the solid state by incorporating the chromophore into polymer films. By appropriately engineering the base molecular structure, the widest range of lasing wavelengths observed for a conventional gain medium can be achieved, in turn establishing a possible route toward high-efficiency light emitters and lasers with near-perfect chromaticity.

## Controllable chromophores for multicolored light emission

A molecule capable of fluorescence and lasing over multiple wavelengths in the visible spectrum and beyond could have applications from sensors and biomarkers to holographic projectors. Organic molecules called chromophores determine which frequencies of light are absorbed and emitted, thus defining a molecule’s color. Alkiviathes Meldrum at the University of Alberta in Edmonton, Canada, and co-workers have engineered a chromophore that emits different colors of light depending on which chemical it is placed in. The team carefully manipulated the base structure of the chromophore to ensure it was soluble and had a wide emission band. In one solvent the chromophore emitted red light, while in another the fluorescence was deep blue. Other chemicals resulted in many other colors, all emitted from a single chromophore. Their design could lead to new types of tunable lasers.

## Introduction

Achieving widely tunable light emission from a single substance has implications for biological imaging, fluorescence- or laser-based sensing, and realizing white-light emitters and lasers. There is a concerted effort toward the development of color-controlled light emission based on single-material systems for these applications, including quantum dots^1,2^, doped fibers^3^, monolithic semiconductors^4^, and microdroplets^5^. Organic chromophores constitute another tunable gain medium that offers exceptional structural and chemical control^6–9^, although they traditionally yield restricted fluorescence shifts and the lasing spectrum is usually limited to within a few tens of nm. Here we show that, by appropriately engineering the chromophore molecular structure, one can design extensive fluorescence control and the widest lasing color gamut obtained from any single emitter.

The ability to extensively tune the wavelength suggests a possible “one-pot” option for mixed-color lasers, sensors, and biomarkers. For example, emitters whose color is especially sensitive to the chemical nature of the environment could offer an improvement over conventional materials used for fluorescence-based sensors and biological imaging. Appropriate wavelength selection can also increase the attainable color space^10^ for high-power illumination sources^11^, holographic projectors^12^, IMAX (trademark name) screens^13^, and headlights^14^. Organic chromophores are often incorporated into organic light-emitting devices^15,16^, but achieving the desired color control requires the use of mutually compatible chromophores, solvents, and polymer hosts, significantly increasing the synthesis complexity^17^.

One way to change the emission spectrum is to chemically alter the base structure of the chromophore to create derivative structures with modified energy levels^18^, but this offers limited wavelength control and relies on the synthesis of fundamentally different compounds. Another method is to use the solvatochromic effect; for example, the fluorescence maximum of 4-phenylumbelliferone, a blue-emitting coumarin derivative, can be shifted by changing the solvent from dimethyl formamide (DMF) to alkaline water^19^. Rhodamine B and polyvinyl diphenylquinoline are tunable over tens of nm by changing the local pH^20^. 7-Hydroxy isoflavone can be tuned over approximately 35 nm in the blue–green region depending on the pH of the solution^21^. Exciplex structures formed from a coumarin derivative might lase over a range of wavelengths by adjusting the solution pH^22^. A lasing color range between orange and red was recently achieved in dicyanomethylene-doped polymers via energy transfer from a conjugated polymer host^23^. In an alternative approach, broad spectrum luminescence was recently reported from the whispering gallery modes of conjugated polymer microspheres^24^.

**Fig. 1:**
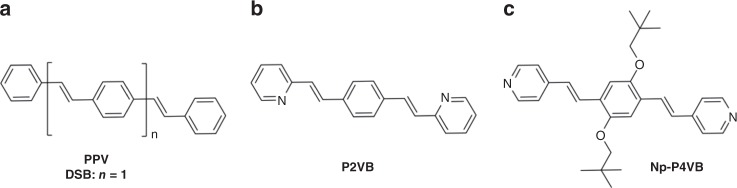
Molecular sketches. Chemical structures of **a** DSB (PPV), **b** P2VB, and **c** Np-P4VB. The key change between DSB and P2VB is the pyridine ring on each end. The difference between the P2VB and P4VB structures is the position of the nitrogen in the pyridine rings. The Np-P4VB synthesized here is similar to P4VB but with the addition of branched neopentyloxy groups on the central benzene ring

Distyrylbenzenes (DSBs; Fig. 1) are a class of fluorescent-conjugated organic molecules that provide structurally defined motifs for poly(phenylene vinylene)s (PPVs)^25^. They can be functionalized by the introduction of different substituents, heteroatoms, or functional groups at desired positions^26,27^ to achieve specific structure–property relationships^28^. Electroluminescent devices^29,30^ and coordination frameworks^31–33^ have been recently fabricated using this class of materials. DSBs can also show amplified spontaneous emission and lasing both in solution and in the solid state^34–38^, and in some cases, they may respond to protonation by shifts in the absorption, fluorescence, or lasing spectra^39–42^. P2VB (1,4-bis(β-pyridyl-2-vinyl)benzene) is a DSB derivative with pyridine groups (Fig. 1b) that tend to interact with the surrounding electronic structure. P4VB (1,4-bis(4-pyridyl-2-vinyl)benzene) has nitrogen atoms on the more accessible fourth ring position, but it appears to associate more readily, and, like P2VB, it has limited solubility in organic solvents^40^.

The addition of specific side groups to the molecular base structure might, in principle, overcome the solubility problem and could limit undesirable intermolecular interactions that decrease the efficiency of conventional chromophores. Here, we place relatively bulky and unreactive neopentyl side groups onto the P4VB base structure to form bis(4-pyridyl)dineopentoxyl-p-phenylenedivinylene (Np-P4VB; Fig. 1c). One may thus, in principle, combine high solubility, a high absolute quantum yield (AQY), and sterically accessible heterocyclic nitrogen atoms on the terminal pyridines to achieve wideband emission color control. Minor changes to the side groups could include carboxyl, amine or other groups aimed toward bioconjugation while maintaining many of the other beneficial features demonstrated below.

## Results

**Fig. 2:**
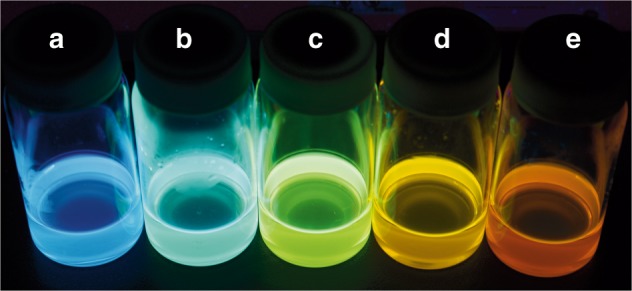
Color photograph of solutions. Photograph of the Np-P4VB chromophore (2 mM except 0.7 mM in cyclohexane) in solution taken under a black light **a** dissolved in cyclohexane (blue); **b** dissolved in DMF (teal); **c** dissolved in DMF with the addition of Zn(NO_3_)_2_ (green); **d** dissolved in DMF with the addition of Hg(NO_3_)_2_ (yellow); and **e** dissolved in acidified DMF (red–orange)

The compound Np-P4VB was synthesized as described in the experimental section. The ^1^H nuclear magnetic resonance (NMR) and electrospray ionization (ESI) mass spectrometric spectra (Fig. S1-S5) as well as the elemental analysis unambiguously verified the chemical structure shown in Fig. 1c. Crystallized from methanol, Np-P4VB formed agglomerations of long, prismatic needles (Fig. S6), giving a yellowish–green fluorescence that peaked at approximately 550 nm. When dissolved at a concentration of <2 mM in cyclohexane, Np-P4VB instead took on a deep blue fluorescence; dissolved in DMF, the fluorescence appeared teal in color. The addition of other soluble impurities, such as metal nitrates or acids, caused the chromophore to undergo a further dramatic color shift toward the green, yellow, or red regions (Fig. 2).

The Np-P4VB featured two absorption bands at wavelengths depending on the solvent. The short-wavelength absorption band (peak 1) could be shifted from 324 to 366 nm, whereas the longer-wavelength band (peak 2) ranged from 393 to 460 nm (Fig. S7; Table 1). For acidified (red–orange fluorescent) solutions, the concentration-dependent changes became saturated at a 1:2 molar ratio of Np-P4VB to H^+^, suggesting that double protonation (i.e., one for each pyridine endmember) completes the effect. Significantly higher concentrations were required for other dissolved ions to achieve saturation, likely due to the reaction kinetics and equilibria that limit the concentration of the dissociated species.

**Table 1:** Summary of Np-P4VB absorption and emission properties

Emission color	Absorption peak (nm)	PL peak (nm)	AQY	*τ* (ns)	*P*_Th_ (μJ/cm^2^)
Blue	324, 393	451	68%	1.88	240
Teal	330, 403	475	82%	2.48	120
Green	—, 419	537	49%	3.09	320
Yellow	—, 430	570	9%	1.54	—
Red	366, 460	591	25%	2.83	330

The first absorption peak in the green and yellow emission was overwhelmed by solvent absorption

**Fig. 3:**
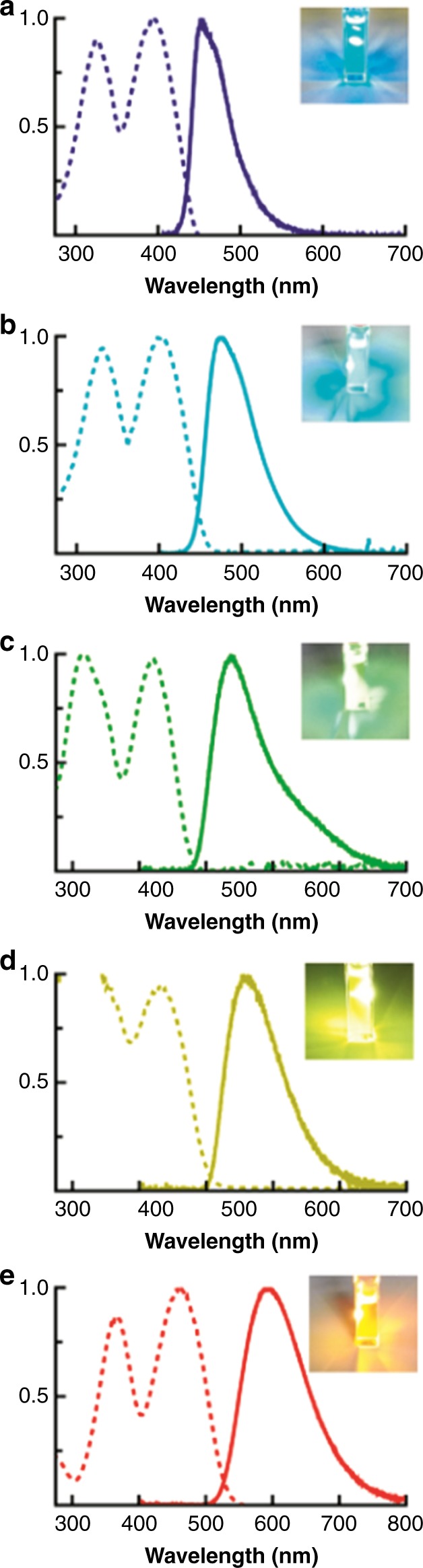
Fluorescence and absorption spectra. Absorption (dashed) and photoluminescence (solid) spectra of Np-P4VB in **a** cyclohexane, **b** pure DMF, **c** DMF with 500 mM Zn(NO_3_)_2_, **d** DMF saturated (~100 mM) with Hg(NO_3_)_2_, and **e** DMF with 500 mM HCl. The concentration of the chromophore for absorption measurements was 0.1 mM, while that for PL measurements was 2 mM (0.7 mM for cyclohexane). The insets show photographs of the luminescence corresponding to the spectrum for each panel

The pure chromophore dissolved in cyclohexane was characterized by broad, asymmetric fluorescence peaking at 451 nm (Fig. 3a; blue line). In DMF, the fluorescence peaked at 475 nm (Fig. 3b; teal line). Upon addition of Zn(NO_3_)_2_ to a chromophore–DMF solution, the peak of the emission spectrum shifted to 537 nm (Fig. 3c; green), while dissolution of Hg(NO_3_)_2_ resulted in a fluorescence at 570 nm (Fig. 3d; yellow). Finally, the addition of an acid (e.g., hydrochloric, acetic, or nitric acid) led to red–orange emission that peaked at 591 nm (Fig. 3e). The fluorescence AQY was 68%, 82%, 49%, 9%, and 25% for the blue, teal, green, yellow, and red Np-P4VB solutions, respectively (Table 1).

**Fig. 4:**
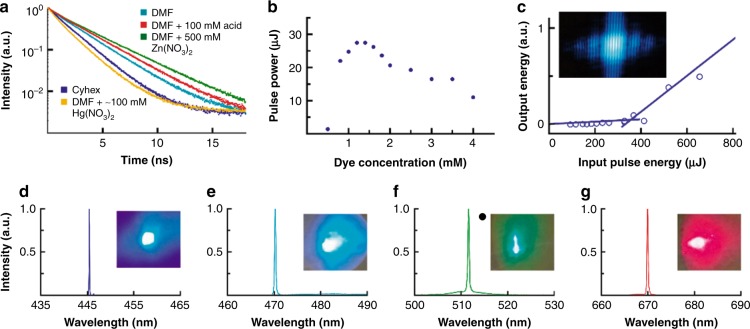
Fluorescence and lasing data. **a** Lifetimes of pure, HCl (acid)-doped, and Zn(NO_3_)_2_-doped solutions of Np-P4VB (3 mM). **b** Output pulse power for various concentrations of Np-P4VB in DMF. **c** Threshold behavior of a 2 mM solution of Np-P4VB in DMF when stimulated at 337 nm by an N_2_ laser. The inset shows a double-slit interference pattern from the laser emission. **d**–**g** show the Np-P4VB laser emission wavelengths for the blue (0.7 mM Np-P4VB in cyclohexane), teal (2 mM Np-P4VB in DMF), green (2 mM Np-P4VB/DMF with 500 mM Zn(NO_3_)_2_), and red lasing (2 mM Np-P4VB/DMF with 0.1 M HCl) solutions

The fluorescence lifetime, *τ*_PL_, for each Np-P4VB solution exhibited a single-exponential decay (Fig. 4a) with a time constant of 1.5 to 3.1 ns (Table 1). The radiative lifetime *τ*_R_ can be estimated for a two-level system (or one in which other transitions such as *S*_2_ → *S*_1_ are fast) with *τ*_R_ *=* *τ*_PL_*/AQY*, yielding 2.76, 3.02, 6.31, 17.11, and 11.32 ns for the blue, teal, green, yellow, and red emission (i.e., generally increasing with emission wavelength), respectively.

## Discussion

Since the emissive species is the same chromophore in each case, the variation in the fluorescence lifetimes can be expected on the basis of the increased mode density at shorter wavelengths according to Fermi’s golden rule, possibly mediated by solvent effects, such as refractive index or viscosity (for example, the visibly higher viscosity of the “nitrated” or acidified solutions could increase *trans-cis* photoisomerization-mediated lifetimes by restricting molecular rotation^43^). There was no appearance of a second lifetime component characteristic of exciplex formation. In contrast to similar compounds without the designed side groups^44^, these results suggest that the luminescence of Np-P4VB is essentially excimer-free single-molecule emission since there was no evidence of additional lifetime components or any significant concentration-dependent luminescence shifts. The excitation for the AQY measurements was the same in every case (405 nm), which aligns with the first absorption band of the blue and teal samples but was located between the emission bands for the other samples, thus likely populating different excited states and leading to different AQYs. For Hg(NO_3_)_2_ in particular, it was difficult to prevent crystallization even at fairly low concentrations; thus the especially low AQY in this case might be due to scattering from microcrystals in solution^45^.

Two absorption bands are a common feature of many conjugated organic chromophores and are likely related to two spin-singlet states, *S*_1_ and *S*_2_, where absorption and emission occur through the usual continuum of vibrational levels. The redshift upon the addition of electron-withdrawing moieties can occur as a result of their interaction with the pyridine end groups. Indeed, protonation of DSB derivatives leads to an increased delocalization of the lowest unoccupied molecular orbital across the whole molecule, which results in a lowering of the excited state energy^41,46–48^. This process should also occur in Np-P4VB to a degree depending on the electron-withdrawing ability of the additive.

As predicted, the emission color of Np-P4VB can be regulated by changing the electron-withdrawing character of the solution, with the additional benefit of its extensive solubility and resistance to exciplex formation. As the solvent becomes more polar or Lewis-acidic, the electrons become more strongly delocalized, causing a decrease in emission energy. Thus, upon transitioning from cyclohexane to DMF, the polarity index increases from 0.2 to 6.4, and the fluorescence shifts from blue to teal. Adding Zn(NO_3_)_2_ or Hg(NO_3_)_2_ increases the Lewis acidity and shifts the emission to green or yellow, respectively. The addition of an acid (hydrochloric acid (HCl), HNO_3_, or CH_3_COOH) leads to an even stronger effect, with the emission becoming orange or red. The fluorescence of Np-P4VB can therefore be tailored or designed throughout the visible spectrum simply by changing the chemical surroundings.

These combined properties suggest that this chromophore may be an excellent color-controlled lasing candidate. Clear evidence of lasing appeared in the emission spectra under 337-nm pulsed excitation in a laser cavity (Fig. 4e, f). For 2 mM Np-P4VB solutions (0.7 mM for cyclohexane), the output pulse powers under nominal 1650 μJ excitation pulses were 400, 286, 123, and 30 μJ for blue, teal, green, and red laser emission, respectively. Only the yellow solution did not lase due to a combination of low AQY and the formation of microcrystals and eventual precipitation at relatively low concentrations. The lasing was characterized by an intense emission above a pump threshold (Fig. 4c) of ~115–130 μJ/cm^2^ (from measurements on new solutions taken several months apart), a linewidth limited by the measured spectrometer response function (63 pm), and a beam divergence of 4.0 mrad. The optimum chromophore concentration to achieve the highest intensity teal output pulses was 1.4 mM (Fig. 4b). At lower concentrations, the decreasing amount of gain medium increases the threshold, whereas at higher concentrations, both increased absorption/scattering and intermolecular interactions typically decrease the output power of organic chromophores^49,50^. The fact that the lowest threshold was obtained for lasing in the teal region may be due to an especially good match between the absorption and pump laser emission for that case.

**Fig. 5:**
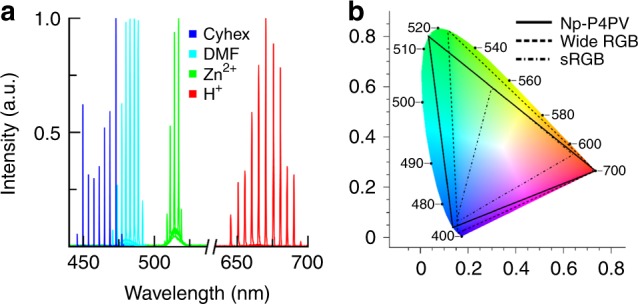
Lasing color range. **a** Lasing wavelengths for Np-P4VB in DMF achievable by tuning the laser cavity. The lasing intensity was normalized across the different groups for ease of visualization. **b** CIE 1931 chromaticity diagram showing the achievable color space from the Np-P4VB laser tuned optimally for 442, 514, and 695 nm, compared with the (Adobe) wide-gamut RGB and the standard RGB color spaces. The unitless *x* and *y* axes are defined as usual in the CIE system

In addition to this wide lasing color range, Np-P4VB is also tunable within each color band by changing the cavity resonant wavelength (Fig. 5a). The tuning ranges for the various color bands are 442–477 nm for blue, 465–495 nm for teal, 505–514 nm for green, and 640–705 nm for red, representing the widest in situ color range so far reported for any single laser gain medium. Moreover, if the Np-P4VB lasing is tuned to 442 nm for blue, 514 nm for green, and 700 nm for red, a large range of the Commission Internationale de l'Éclairage (CIE) color space is enclosed (Fig. 5b). The color space achievable from lasing Np-P4VB is much greater than that of the standard red, blue, green (sRGB; 39.5% coverage) and has an area equivalent to that of Adobe’s wide-gamut RGB color space (77.6% coverage).

What gives Np-P4VB such a huge emission range of the visible color spectrum? One key aspect is the addition of neopentyl sidechains to the P4VB base molecule, thus sterically shielding the conjugated molecular backbone. This both increases its solubility up to 94 mM in DMF (19 mM in ethanol) and prevents aggregate formation. Aggregation leads to intermolecular exciplex formation, causing a transition to dimer emission, which can severely limit the achievable lasing wavelengths. An obvious additional benefit of the P4VB molecule lies in its end group reactivity. While in this work we demonstrated only RGB lasing within a set of wavelength ranges for each color, we believe that designing the Lewis acidity of the solvent could allow Np-P4VB or other similarly engineered chromophores to fluoresce or lase at virtually any wavelength in the visible spectrum and beyond.

**Fig. 6:**
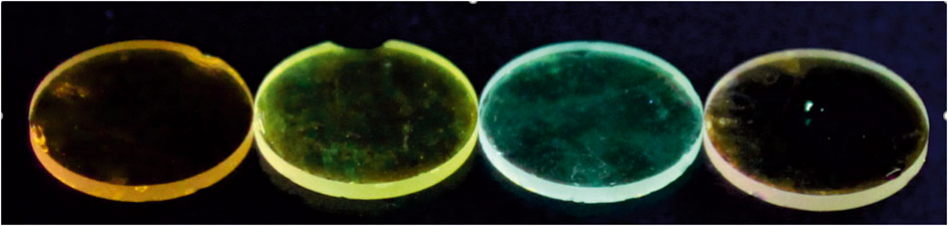
Photograph (raw unprocessed data) of red–orange, yellow–green, teal, and white polyelectrolyte bilayer films containing dissolved Np-P4VB. A hand-held overhead blacklight was used for excitation. The corresponding PL spectra are shown in Fig. S8. The wafers are 1 cm in diameter

There are several advantages of a single wide-spectrum lasing compound. Incorporating the various solutions into a polymer or polyelectrolyte (PE) would permit fluorescence (and possibly lasing) with extensive color control, presenting a simplification over the processing required for different chromophore–polymer mixtures. For imaging and sensing applications, a solution-based and widely tunable fluorescent or lasing medium is of course of interest; however, for most light emission applications one needs a solid medium. Here we demonstrate color-controlled molecular layers emitting over a range of colors including white (Fig. 6). In each case, the emissive region is a Np-P4VB-doped monolayer of polyallylamine hydrochloride (teal) or polystyrene sulfonate (green–yellow and red–orange; see experimental). Stacking these layers allows a wide gamut of emissive color to be obtained, which is controlled by the number of layers of each color. These results imply that white emission, or indeed almost any color, can be achieved in solid as well as in liquid media using a single emissive chromophore.

This work demonstrates extensive lasing color control over a range of wavelengths that can encompass almost the entire CIE color space. The protective side groups and accessible pyridine end groups can combine good absolute quantum efficiency, high solubility, and minimal intermolecular interactions while permitting a large degree of energy level control. The same concepts could almost certainly be applied to other organic systems to engineer massively tunable fluorescence and lasing. Of course, organic chromophores, especially PPV-based polymers, can have problems with photooxidation and triplet formation, which implies that the lasing will degrade over time. No noticeable degradation was observed in the solution samples after 30 min of lasing (at 10 Hz), but highly viscous solutions made by the addition of polymers did show photobleaching and quenching of the lasing output. However, these issues might be minimized via the addition of auxiliary compounds that block triplet formation, as shown for related PPV structures^51^.

In summary, we demonstrate a molecular engineering blueprint for designing chromophores featuring high solubility, high quantum efficiency, and a molecular structure that allows unprecedented energy-level tunability via solvent interactions. The designed chromophore chosen to illustrate these concepts lases across almost all of the visual color spectrum as a consequence of simple Brønsted/Lewis acid–base interactions. Only complex and expensive nonlinear devices such as parametric amplifiers can currently match this method for such extensive laser wavelength control.

## Materials and methods

All solvents and reagents used in the synthesis were purchased from commercial sources and were applied without additional purification unless stated otherwise.

### Synthesis of Np-P4VB

**Fig. 7:**
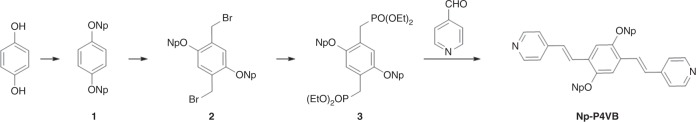
Scheme showing the intermediate products during the synthesis of Np-P4VB. The products are numbered according to the step of the synthetic route. Step 4 shows the Wittig–Horner-type reaction, which produces Np-P4VB

Np-P4VB was prepared according to the four-step route shown in Fig. 7.

Step 1:

*1,4-dineopentyloxy benzene (1)* was first synthesized by a modification of a procedure described by *Quast* et al.^52^. Hydroquinone (1.10 g; 9.99 mmol; 1.0 eq.) together with cesium carbonate (16.02 g; 45.40 mmol; 4.5 eq.) were placed in a flame-dried pressure *Schlenk* flask, evacuated, and flooded with argon. Neopentyl bromide (3.43 mL, 4.11 g, 27.25 mmol; 2.8 eq.) was degassed and dried over molecular sieves, then *N*-methylpyrrolidon (NMP, 30 mL) was added. The reaction mixture was gently evacuated, heated to 160 °C, and stirred for 3 days. After cooling to ambient temperature, the reaction mixture was poured into 700 mL of ice-cold water, and the colorless precipitate was filtered after 15 min of stirring. Recrystallization from ethanol yields compound 1 as colorless needles (2.48 g, 9.90 mmol; 99%).

Step 2:

*1,4-bis(bromomethyl)−2,5-bis(neopentyloxy) benzene (2)* was synthesized via a Blanc-type bromomethylation of 1, similar to a recently described procedure^53^. Compound 1 (3.82 g; 15.26 mmol; 1.0 eq.) and paraformaldehyde (1.01 g; 33.57 mmol; 2.2 eq.) were evacuated, flushed with argon, and then suspended in acetic acid (100 mL). A hydrogen bromide solution in acetic acid (30%; 9.05 g; 33.57 mmol; 2.2 eq.) was added. After stirring for 15 min at ambient temperature, the suspension was further stirred at 65 °C overnight. After cooling to room temperature, the resulting yellow-brownish suspension was poured into ice-cold water (650 mL), stirred for 10min, and the white precipitate was filtered off. The raw product was dissolved in hot chloroform (20 mL) and precipitated by the addition of methanol (180 mL). The flask was slowly cooled to 5 °C and stored overnight at this temperature. After filtration, the solution was concentrated to 50% of its volume and again cooled to 5 °C and filtered. The collected solid fractions were dried under reduced pressure yielding product 2 (3.84 g; 8.80 mmol; 58%) as fine colorless needles.

Step 3:

*1,4-bis(diethylphosphonatomethyl)−2,5-bis(neopentyloxy) benzene (3)* was synthesized using the method established by Iwase et al.^54^. The bromomethyl derivative 2 (0.15 g; 0.34 mmol; 1.0 eq.) was placed in a *Schlenk* flask under argon. After the addition of triethylphosphite (2.4 mL; 2.28 g; 13.76 mmol; 40 eq.), the mixture was stirred at 150 °C overnight. The clear solution turned into a white suspension after cooling to ambient temperature. Residual triethylphosphite was removed via vacuum distillation. The solid crude product was vigorously stirred in 3 mL of water until a homogeneous suspension formed. After filtration and drying on air, product 3 (0.18 g; 0.32 mmol; 84%) was obtained as a colorless solid.

Step 4:

*2,5-Bis(2-(4-pyridyl)-vinylene) hydroquinone dineopentyl ether (Np-P4VB)* was finally synthesized following a Wittig–Horner-type reaction adapted from the methyl derivative^54^. In a preheated *Schlenk* flask, phosphonate 3 (0.19 g; 0.34 mmol; 1.0 eq.) and potassium *tert*-butoxide (0.12 g; 1.04 mmol; 3.0 eq.) were dissolved in dry *tert*-butanol (10.0 mL). To the resulting yellow-greenish mixture, a solution of 4-formyl pyridine (0.10 mL; 0.12 g; 1.07 mmol; 3.1 eq.) in *tert*-butanol (1 mL) was added dropwise. During this addition, the solution turned brown and became fluorescent. The solution was then stirred at 60 °C overnight and subsequently poured into ice-cold water (100 mL). After filtration, the resulting solid was washed with 10 mL of water three times and then recrystallized from methanol to yield Np-P4VB (0.08 g; 0.18 mmol; 52%) as long needles exhibiting yellow fluorescence.

### Preparation of dye solutions

Finally, the purified Np-P4VB was dissolved in DMF for optical characterization. The concentrations varied from 0.05 mM to 4 mM with a measured saturation of 94.1 mM. To modify the electronic structure and optical properties of Np-P4VB via protonation, we acidified the solutions by adding defined amounts of aqueous HCl solutions varying from 0.001 to 12 M. To achieve the changes in electronic properties via the interaction with zinc ions, we added pure Zn(NO_3_)_2_ salt to concentrations varying from 1 to 3000 mM. Zn^2+^ was chosen as a solution dopant because it is not paramagnetic; therefore, laser quenching is minimized, and previous work has shown that the pyridinyl nitrogens chelate with zinc ions to form coordination frameworks^31^.

### NMR spectroscopy and other analytics

NMR spectroscopy was applied to confirm the structure of the intermediate products and of the goal compound Np-P4VB. ^1^H NMR spectra were measured on a Bruker AV-III 300 MHz spectrometer using deuterated chloroform as the solvent for the intermediate products. Np-P4VB was analyzed in CD_2_Cl_2_ to avoid overlapping of the residual solvent peak with the signals of aromatic protons of the compound. The signals in all spectra were referenced to the residual solvent peak (7.24 ppm for chloroform and 5.30 ppm for CHDCl_2_). The recorded spectra are in complete agreement with the respective structures shown in Fig. 7. The details on the signal assignment are given in supplemental information.

The structure of compound Np-P4VB was additionally confirmed by ESI mass spectroscopy using a Varian LC MS 500. Protonated molecular ion and doubly protonated molecular ion peaks along with a Na^+^-adduct molecular peak were observed using acetonitrile (LC-MS grade) as the solvent. The C, H, and N elemental composition of Np-P4VB was determined using either EA Euro 3000 (Kehatech) or Elementar Vario EL. The results are in good agreement with the values calculated for the structure.

### Preparation of emissive PE films

PE films were made from Np-P4VB (1 mM) dissolved in polyallylamine hydrochloride (PAH) dissolved in water (10 or 20 mg/mL) or polystyrene sulfonate (PSS) dissolved in water (20 mg/mL). Higher dye concentrations were also tested, but these tended to lead to precipitation (cloudy solutions) and/or the formation of rough films. Lower concentrations worked fine in terms of film quality but were obviously less luminescent. We then followed a recipe similar to that developed in ref. ^55^ for monolayer PE films. A drop of PAH solution (20 mg/mL) was dropped onto a quartz wafer that was initially cleaned with concentrated NaOH solution. The droplet was immediately washed in pure water to leave a single PE layer bound to the surface. The orange films were produced by adding HCl to the PSS solution to a concentration of 0.01 M (PSSo); the green ones were prepared similarly but using Zn(NO_3_)_2_ (0.032 mM) instead of HCl (PSSg). To produce the blue layers, NaOH was added to a PAH stock solution to a concentration of 0.01 M (PAHb). The blue fluorescent sample consisted of PAH-(PSS-PAHb)×3; the green was (PAH-PSSg)×3, and the orange was (PAH-PSSo)×3. Finally, the white film consisted of PAH-(PSS-PAHb)×4-PSS-(PAH-PSSo)×2-(PAH-PSSg)×2.

### Absorption and photoluminescence (PL) spectroscopy

Absorption measurements were performed using a Hewlett-Packard 8452A Diode Array spectrophotometer, using a cuvette filled with pure solvent as the blank. For PL spectroscopy, we used the 405-nm ion line of a diode laser operated at an excitation power density of ~1250Wm^-2^ as the excitation source. The PL was collected by an optical fiber (numerical aperture ~0.22) and directed to an Ocean Optics USB 2000+ miniature spectrometer. The efficiency of the system was corrected with an Ocean Optics calibrated light source. Photographs of the PE layers were taken with a Canon Rebel T3i camera.

### PL dynamics

PL decay traces were taken using an Alphalas picosecond pulsed laser at a wavelength of 405 nm and nominal pulse length of 40 ps. The PL was collected using a fiber optic system, passed through a longpass filter to remove scattered excitation light, and directed into a Becker-Hickl HPM-100-50 photomultiplier tube with a response time of ~100 ps. The decay traces were obtained using the SPC-130 module from Becker-Hickl.

### Absolute QY

AQY measurements were performed using an adapted integrating sphere. Blank or sample solutions were placed into a cuvette that was lowered into the integrating sphere on a magnetic holder. A 405 nm light emitting diode was used as the excitation source. The PL and excitation intensities were captured through a fiber attached to the sphere and analyzed with a Ocean Optics spectrometer and a calibrated light source. “Blank” measurements were taken with a cuvette filled with DMF only, whereas “sample” stands for the measured intensities with the dissolved sample present in the solution.

### Lasing

**Fig. 8:**
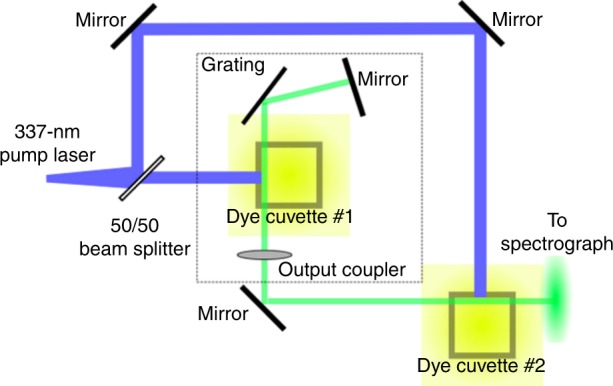
Simplified diagram of the laser cavity set-up. Cuvette #1 is placed in the tunable lasing cavity and is excited with 337-nm pulses from a nitrogen laser. The lasing output is directed through cuvette #2 for further amplification before entering the spectroscopy system

Lasing from Np-P4VB dissolved in DMF was characterized using the double-cuvette laser cavity inside a PTI GL-302 dye laser. The first cuvette is contained in the main tunable laser cavity, while the second identical solution is used to further amplify the output (Fig. 8). The dye laser cavity was pumped using the 337 nm output of a GL-3000 nitrogen laser operated at 3–4 Hz, with 1 ns pulses having an energy of ~1 mJ. The Np-P4VB lasing output power was measured using a Gentec-EO Solo 2 monitor with an XLP12 thermopile protected by an aluminum draft shield, which was allowed to stabilize for 10 min before taking measurements.

The sample emission, if present, was attenuated using a neutral density filter or a set of polarizers and sent to the Ocean Optics miniature spectrometer calibrated as described above. The spectrometer response function was measured to be 5 nm using the 442 nm line (bandwidth ~3 GHz) of a HeCd gas laser. Higher-resolution measurements were taken using a Santa Barbara Instruments Group ST-8 spectrometer with a tunable grating. The response function of this system was 63 pm, which was measured in the same way.

## Electronic supplementary material

Supplemental Information
