# Supplementary material for: Wide-gamut lasing from a single organic chromophore
Source: Light Sci Appl. 2018 Dec 5;7:101. doi: 10.1038/s41377-018-0102-1 (PMC6279737; doi:10.1038/s41377-018-0102-1)
Supplement: Supplementary file 1 — Supplemental Information [file 41377_2018_102_MOESM1_ESM.docx]

Wide-gamut RGB lasing from a single organic chromophore

S. Lane^^[[1]](#footnote-1)^^, S. Vagin^^[[2]](#footnote-2)^^, H. Wang^1^, W. Heinz^2^, W. Morrish^1^, Y. Zhao^1^, B. Rieger^2^, and A. Meldrum^1^

**Supplemental Information**


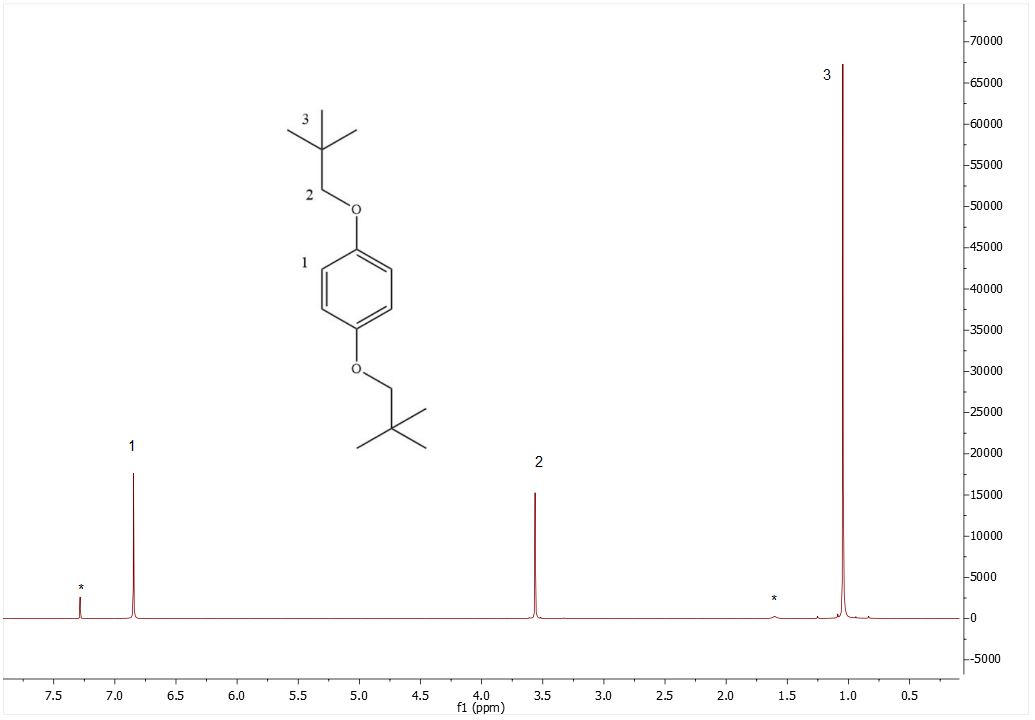


Fig. S1. Compound 1: ^1^H-NMR (300 MHz, CDCl_3_, 300 K): δ (ppm) = 6.85 (s, 4H, Harom), 3.56 (s, 4H, -CH_2_), 1.04 (s, 18H, -CH_3_). Signals of solvent impurities (CHCl_3_, H_2_O) are marked with asterisk.


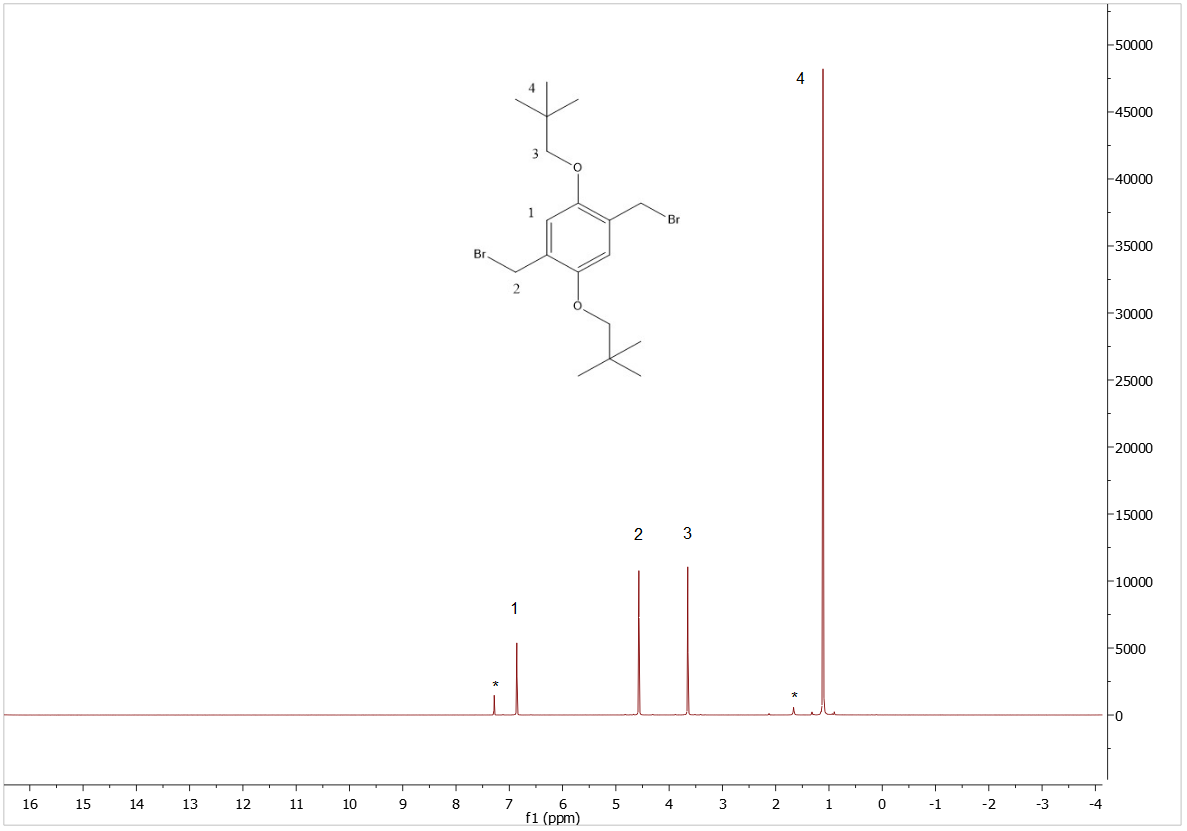


Fig. S2. Compound 2: ^1^H-NMR (300 MHz, CDCl_3_, 300 K): δ (ppm) = 6.86 (s, 2H, Harom), 4.57 (s, 4H, -CH_2_Br), 3.65 (s, 4H, -OCH_2_), 1.11 (s, 18H, -CH_3_). Signals of solvent impurities (CHCl_3_, H_2_O) are marked with asterisk.


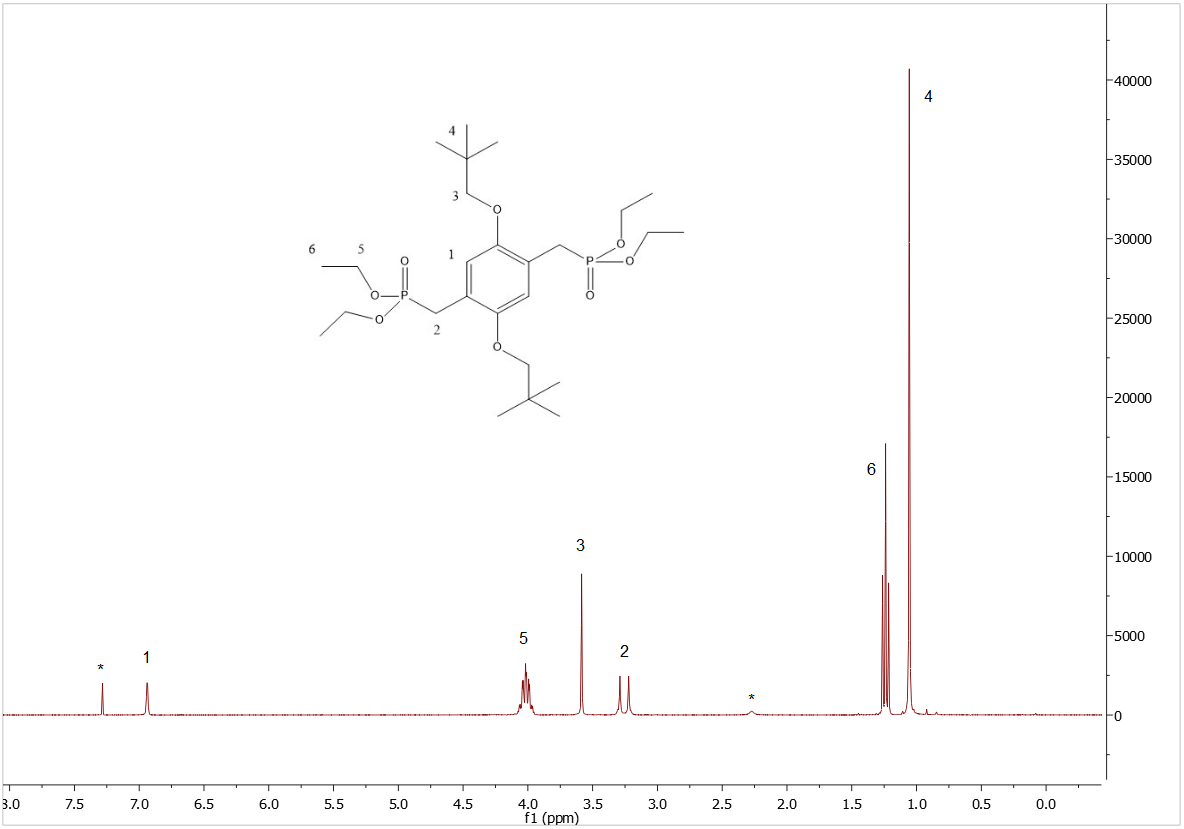


Fig. S3. Compound 3: ^1^H-NMR (300 MHz, CDCl_3_, 300 K): δ (ppm) = 6.95 (s, 2H, *H*_arom_), 4.09 – 3.96 (m, 8H, -OCH_2_CH_3_), 3.59 (s, 4H, -OCH_2_), 3.26 (d, *^2^J_H-P_* = 20.3 Hz, 4H, -C*H*_2_P), 1.25 (t, *^3^J_H-H_* = 7.1 Hz, 12H, -OCH_2_CH_3_), 1.06 (s, 18H, -CH_3_). Signals of solvent impurities (CHCl_3_, H_2_O) are marked with asterisk.


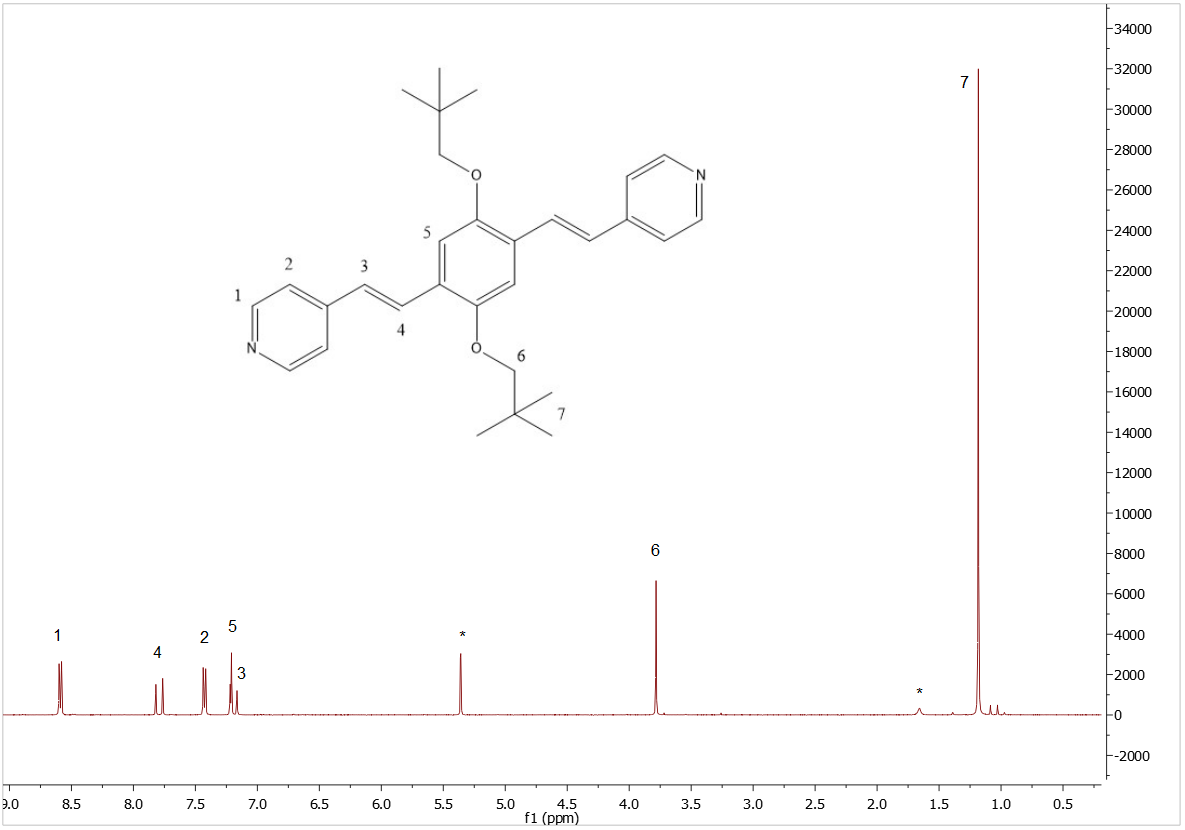


Fig. S4. Compound Np-P4VB: ^1^H-NMR (300 MHz, CD_2_Cl_2_, 300 K): δ (ppm) = 8.55 (d, *^3^J* = 6.2 Hz, 4H, py-*H*_ortho_), 7.75 (d, *^3^J* = 16.5 Hz, 2H, inner vinylene *H*), 7.39 (d, *^3^J* = 6.2 Hz, 4H, py-*H*_meta_), 7.17 (s, 2H, -CH_arom_), 7.15 (d, *^3^J* = 16.5 Hz, 2H, outer vinylene *H*), 3.75 (s, 4H, -OCH_2_), 1.15 (s, 18H, -CH_3_). Signals of solvent impurities (CHDCl_2_, H_2_O) are marked with asterisk.


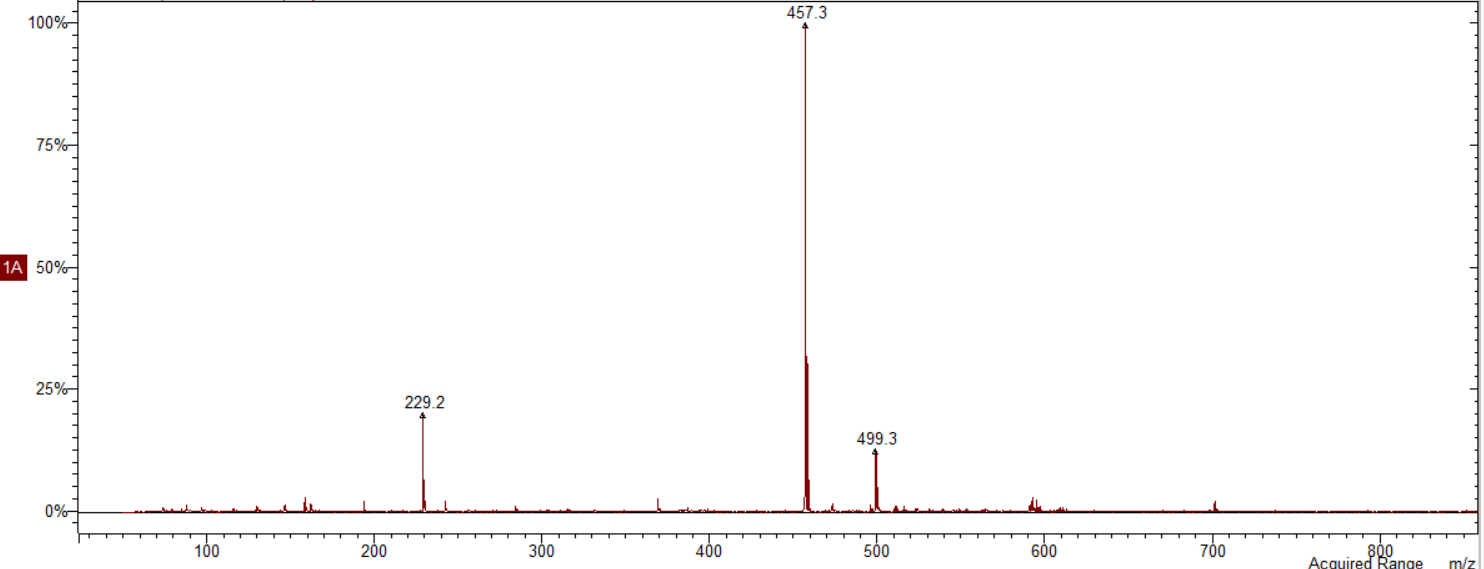


Fig. S5. Compound Np-P4VB: ESI-MS (MeCN): 229 [M+2H]^2+^, 457 [M+H]^+^, 499 [M+Na]^+^.


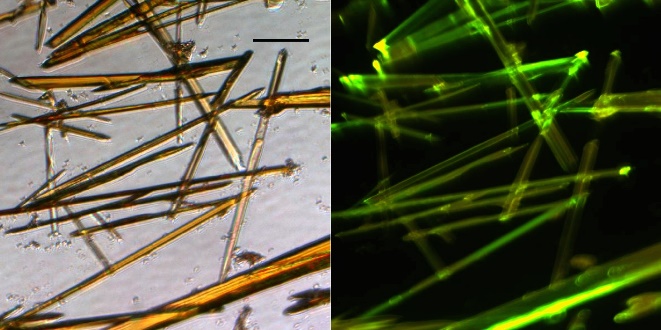


Fig. S6. Crystals of Np-P4VB in transmitted light (left) and fluorescence (right). The scale bar is 100 μm.


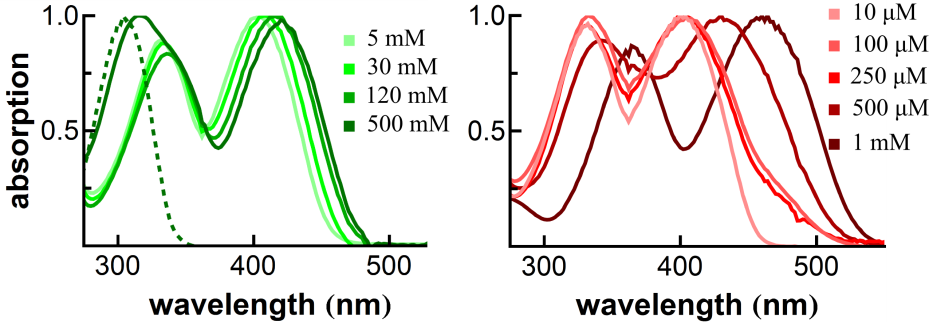


Fig. S7: a) Normalized absorption spectra of 0.07 mM Np-P4VB in DMF containing various concentrations of Zn(NO_3_)_2_. The dashed line shows the absorption of a 500 mM Zn(NO_3_)_2_ solution in DMF. b) Absorption spectra of Np-P4V in DMF with various concentrations of HCl.

*
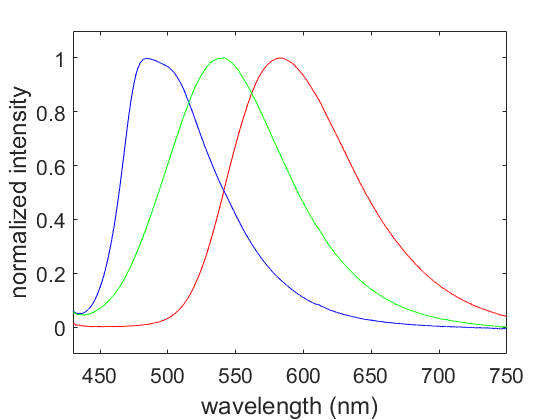
*

Fig. S8: Normalized photoluminescence spectra of the teal, green-yellow, and orange polyelectrolyte films.

1. Department of Physics, University of Alberta, Edmonton, AB, T6G2E1, Canada [↑](#footnote-ref-1)
2. Wacker Chair of Macromolecular Chemistry, Technical University of Munich, Lichtenbergstraße 4, 85747 Garching bei München, Germany [↑](#footnote-ref-2)
